# Supplementary material for: Netcombin: An algorithm for constructing optimal phylogenetic network from rooted triplets
Source: PLoS One. 2020 Sep 18;15(9):e0227842. doi: 10.1371/journal.pone.0227842 (PMC7500971; doi:10.1371/journal.pone.0227842)
Supplement: S4 File — (ZIP) [file pone.0227842.s004.zip › SequenceData-Triplet/sequenceData-10-20-30-0-Triplet/length 400, numseq 40, rate 10^-7/4 seq.pdf]

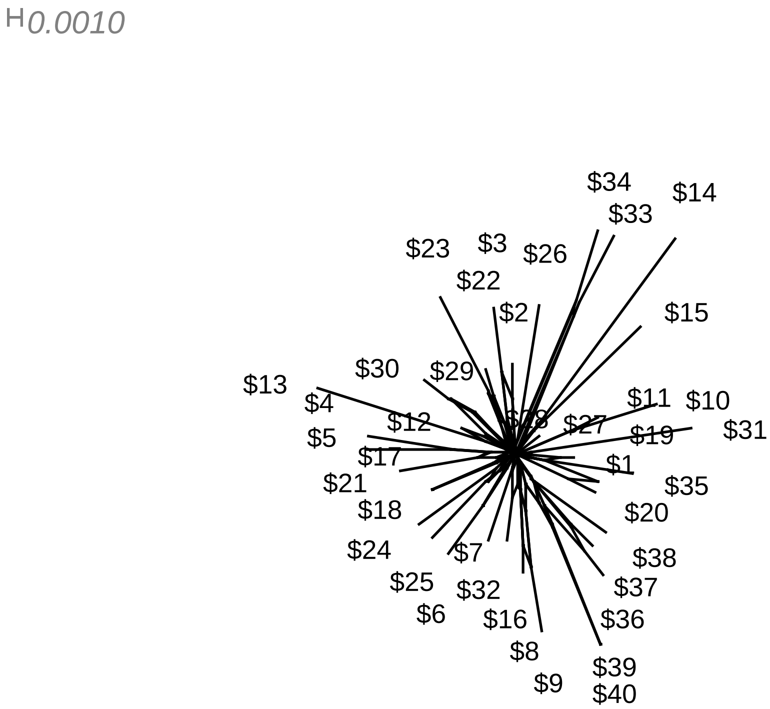
0.0010
$34
$14
$33
$23
$3
$26
$22
$2
$15
$30
$29
$13
$4
$11
$10
$12
$28
$27
$5
$19
$31
$17
$1
$21
$35
$18
$20
$24
$7
$38
$25
$37
$32
$6
$16
$36
$8
$39
$9
$40
